# Integrated metabolomic and transcriptomic analysis reveals digestive tract adaptations to high altitude in Bayanbulak sheep

**DOI:** 10.3389/fvets.2025.1687858

**Published:** 2025-12-01

**Authors:** Xuhui Chen, Bin Chen, Yaling Yang, Lingling Liu, Wujun Liu

**Affiliations:** College of Animal Science, Xinjiang Agricultural University, Ürümqi, China

**Keywords:** Bayanbulak sheep, Turpan black sheep, metabolome, transcriptome, high-altitude adaptation

## Abstract

**Introduction:**

High-altitude adaptation in Bayanbulak sheep may modulate digestive tract functions. This study investigated differences in digestive tract metabolites between Bayanbulak sheep at high altitude (2,400–4,400 m) and Turpan black sheep at low altitude (−154 m). We performed untargeted metabolomics analysis on ruminal fluid, small intestinal contents, and fecal metabolites, measured serum biochemical parameters, and examined skin tissue morphology. Differential gene expression in ruminal fluid was analyzed using RNA-seq. Key findings include: (1) Bayanbulak sheep showed significantly higher including total antioxidant capacity (TAC), superoxide dismutase (SOD), catalase (CAT), interferon-γ (IFN-γ), interleukin-2 (IL-2), total cholesterol, glucose, and triglyceride levels compared to Turpan black sheep (*p* < 0.05); (2) Epidermal thickening was observed in Bayanbulak sheep under high-altitude conditions. (3) Significant upregulation of metabolites including spermidine, 5′-adenylic acid (AMP), oleic acid (OA), and nicotine occurred in Bayanbulak sheep, accompanied by enhanced activity in glutathione metabolism, AMP-activated protein kinase (AMPK) signaling pathway, purine metabolism, and thermogenesis pathways. (4) Co-detected metabolites - vanillin, pinacidil, and 4-hydroxychalcone (4-HC) - exhibited significant differential abundance across rumen, small intestine, and feces in Bayanbulak sheep, with concurrent upregulation of associated metabolic pathways. (5) In the rumen of Bayanbulak sheep, genes CPT1B (carnitine palmitoyltransferase 1B), CPT1C (carnitine palmitoyltransferase 1C), and CASTOR2 (cytosolic aspartate tRNA-specific ribozyme) were significantly upregulated (*p* < 0.05), collectively enriched in the AMPK signaling pathway and mTOR signaling pathway, while the small intestinal gene TM4SF18 (transmembrane 4 L six family member 18) was significantly downregulated (*p* < 0.01), co-enriched in the ferroptosis pathway.

**Conclusion:**

Bayanbulak sheep adapt to high-altitude hypoxia and thermal stress through epidermal thickening and synergistic interactions between blood and digestive tract metabolites. These adjustments enhance antioxidant capacity, energy reserves, and immune function, offering a robust model for high-altitude adaptation.

## 1 Introduction

The Bayanbulak Grassland experiences extremely harsh climatic conditions and is a high-altitude mountainous region situated between 2,400 and 4,400 meters above sea level (1). The area receives snowfall for approximately 160 days annually, with a yearly precipitation of less than 400 millimeters. The mean annual temperature ranges from −5.5 °C to −3.5 °C, forming a typical cold and arid high-altitude climate (2). As altitude increases, the atmospheric oxygen concentration significantly decreases, dropping from 21% at plain elevations to between 16.3 and 12.7% at altitudes of 2,000 to 4,000 meters (3).

In this environment, Bayanbulak sheep have evolved exceptional cold and hypoxia tolerance through long-term natural selection. They efficiently utilize limited resources to sustain themselves in the sparse vegetation of the high-altitude meadows (1). In stark contrast to the Bayanbulak sheep is the Turpan black sheep (also known as the Toksun black sheep). This breed is primarily distributed in the Turpan Basin, the lowest-lying depression in China, with a minimum elevation of −154 meters below sea level (4). As a high-quality indigenous meat breed, the Turpan black sheep has developed unique survival strategies through long-term adaptation to aridity and high temperatures. This low-altitude environment forms a natural comparative system with the high-altitude, hypoxic conditions of the Bayanbulak grassland, providing critical samples for studying animal adaptation under divergent ecological pressures.

Current research indicates that the adaptation mechanisms of ruminants to high-altitude environments exhibit both diversity and species specificity. For instance, Tibetan sheep utilize synergistic interactions between rumen microbiota and host metabolomes to significantly enhance energy metabolic efficiency. Their rumen microbial communities can convert substantial amounts of Nε-fructosyllysine into butyrate, thereby augmenting energy acquisition and supporting hypoxia tolerance (5). Mongolian sheep, in contrast, enhance thermogenic capacity through the browning of white adipose tissue while simultaneously optimizing ruminal nutrient absorption efficiency to cope with cold climates (6). At the genetic level, polymorphisms in the COX17 gene of Tibetan sheep improve oxygen utilization efficiency under hypoxic conditions by regulating mitochondrial copper ion homeostasis (7). These findings collectively demonstrate that high-altitude adaptation results from the integrated effects of metabolism, microbiome function, and genetic regulation.

Building upon this background, this study focuses on a comparative metabolomic analysis of Bayanbulak sheep and Turpan black sheep, aiming to uncover the metabolic principles underlying their environmental adaptations. The research will systematically collect rumen metabolome, small intestine metabolome, and fecal metabolome data across distinct altitudinal gradients (Bayanbulak grassland: 2400–4,400 m vs. Turpan Basin: −154 m). Utilizing multi-omics integration analysis techniques, the study will elucidate the mechanisms by which Bayanbulak sheep maintain energy homeostasis and resist oxidative damage. The findings will not only address the current gap in research on high-altitude cold adaptation in sheep but also provide a theoretical foundation and technical support for ruminant genetic breeding (e.g., breeding hypoxia-tolerant strains) and medical research (e.g., developing treatments for hypoxia-related diseases).

## 2 Materials and methods

### 2.1 Experimental animals and sample collection

This study utilized fifteen healthy, one-year-old Bayanbulak sheep (BY) and fifteen Turpan black sheep (TLF) as experimental subjects. All animals were raised under identical management conditions and were free of disease. The sheep were divided into two groups (*n* = 15 per group). All animals had been vaccinated against ovine caseous lymphadenitis (CLA), Peste des Petits Ruminants (PPR), Foot-and-Mouth Disease (FMD), and sheep pox. Additionally, they were regularly treated with ivermectin and abamectin for the prevention and control of internal and external parasites.

### 2.2 Sampling procedure

At the conclusion of the 120-day feeding trial, all animals were fasted (solid and liquid) for 12 h following animal welfare protocols. They were then transported to a local commercial slaughterhouse and humanely euthanized according to standard procedures (70). Ante-mortem, jugular venous blood was collected into clot activator tubes. Whole blood samples were allowed to clot at room temperature (RT, 18–25 °C) for 15–30 min until a clear, pale yellow supernatant (normal serum) separated. Samples were centrifuged at 3500 rpm for 5 min using a portable centrifuge. Serum was aspirated and aliquoted into sterile microcentrifuge tubes. The collection date, sample volume, and corresponding sheep identification number were recorded. Serum aliquots were stored in a refrigerated chamber (2–8 °C) pending analysis. Following exsanguination and confirmation of unconsciousness, rumen fluid, small intestinal contents, and fecal samples were collected. All samples were immediately placed on dry ice for transport to the laboratory and subsequently stored at −80 °C until further analysis.

### 2.3 Serum biochemistry analysis

All serum samples were analyzed for various biochemical parameters in accordance with the manufacturer’s instructions of the ELISA kit provided by Nanjing Jiancheng Bioengineering Institute. Serum biochemical parameters were analyzed using the Catalyst One chemical analyzer (IDEXX Laboratories, Westbrook, MA, USA). The parameters measured included: Total IFN-*γ* (Interferon-gamma) content (ng/L), TNF-*α* (Tumor Necrosis Factor-alpha) content (ng/L), IL-2 (Interleukin-2) content (ng/L), IgG (Immunoglobulin G) content (mg/mL), Pi (Inorganic Phosphorus) content (mmol/L), Ca (Calcium) content (mmol/L), TC (Total Cholesterol) content (mmol/L), TG (Triglycerides) content (mmol/L), GLU (Glucose) content (mmol/L), *γ*-GT (Gamma-Glutamyl Transferase) activity (U/L), T-BIL (Total Bilirubin) concentration (μmol/L), BUN (Blood Urea Nitrogen) content (mmol/L), UA (Uric Acid) content (μmol/L), Cr (Creatinine) content (μmol/L), AST (Aspartate Aminotransferase) activity (U/L), ALT (Alanine Aminotransferase) activity (U/L), ALP (Alkaline Phosphatase) activity (King Armstrong units/100 mL), CK (Creatine Kinase) activity (U/mL), LDH (Lactate Dehydrogenase) activity (U/L), T-AOC (Total Antioxidant Capacity) activity (mmol/L), CAT (Catalase) activity (U/mL), GSH-Px (Glutathione Peroxidase) activity (U/mL), MDA (Malondialdehyde) content (nmol/mL), SOD (Superoxide Dismutase) activity (U/mL), ALB (Albumin) content (g/L), Total Protein content (g/L).

### 2.4 Integumentary morphological analysis

Fixed skin tissue samples were trimmed flat and dehydrated through an ascending series of ethanol concentrations. Following clearing in xylene, the tissues were embedded in paraffin wax. After the wax solidified, sections were cut at a thickness of 4 μm. Paraffin sections were deparaffinized and rehydrated. They were then stained with hematoxylin for 3–5 min, differentiated in acid alcohol (differentiator), and rinsed in tap water for bluing (10 min). Subsequently, sections were counterstained with eosin for 2 min. Following staining, sections were dehydrated through an ascending ethanol series, cleared in xylene, and mounted with neutral balsam. Sections were observed and photographed under a light microscope.

### 2.5 Metabolomic analysis of rumen fluid, small intestinal contents, and feces

Samples were retrieved from the −80 °C freezer and thawed on ice until no ice crystals remained (all subsequent procedures were performed on ice); after thawing, samples were vortexed for 10 s for homogenization, and 200 μL of each sample was transferred to correspondingly labeled centrifuge tubes, followed by addition of 200 μL of 20% acetonitrile-methanol internal standard extraction solution, vortexing for 3 min, and centrifugation at 12,000 rpm for 10 min at 4 °C; subsequently, 350 μL of supernatant was transferred to new correspondingly labeled centrifuge tubes and concentrated to complete dryness; the dried residues were reconstituted in 100 μL of 70% methanol aqueous solution, vortexed for 3 min, sonicated in an ice-water bath for 10 min, then centrifuged at 12,000 rpm for 3 min at 4 °C, and 80 μL of supernatant was transferred to inserts of correspondingly labeled autosampler vials for instrumental analysis. Liquid chromatography-mass spectrometry analysis was performed using a Waters ACQUITY Premier HSS T3 Column (1.8 μm, 2.1 mm × 100 mm) with mobile phases consisting of 0.1% formic acid in water (A) and 0.1% formic acid in acetonitrile (B), a column temperature of 40 °C, flow rate of 0.4 mL/min, and injection volume of 4 μL. Raw mass spectrometry data were converted to mzML format using ProteoWizard, and peak detection, alignment, and retention time correction were performed using XCMS; peaks with missing values >50% across samples were filtered, and missing values were imputed using KNN imputation + 1/5 minimum value approach (values with >50% missingness were filled with 1/5 minimum value, while those with <50% missingness were filled using KNN), with peak areas normalized using Support Vector Regression (SVR); processed peaks were used for metabolite identification by searching against a laboratory-built database, integrated public databases, predictive databases, and through metDNA analysis; metabolites with identification scores >0.5 and QC sample coefficient of variation (CV) values <0.3 were retained, and peaks from positive and negative ionization modes were merged, keeping the identification with the highest confidence level and lowest CV value; differential metabolites were screened by combining fold change (FC) values, t-test *p*-values, and variable importance in projection (VIP) scores from OPLS-DA models, with thresholds set at |FC| > 1, *p* < 0.05, and VIP > 1, followed by KEGG functional annotation and enrichment analysis of differential metabolites (8).

### 2.6 RNA isolation and sequencing

Total RNA was isolated from rumen and small intestinal tissues of Bayanbulak sheep and Turpan black sheep across distinct geographical regions using TRIzol reagent. RNA concentration and purity were quantified via NanoDrop 2000 spectrophotometry (Thermo Fisher Scientific), while RNA integrity was assessed using an Agilent 2,100 Bioanalyzer. Samples with RNA Integrity Number (RIN) > 8.5 and 28S/18S ribosomal RNA ratio >0.7 were selected for library construction.

Polyadenylated mRNA was enriched by Oligo(dT) magnetic bead purification followed by DNase I treatment. Purified mRNA was fragmented, and double-stranded cDNA was synthesized and purified. After end repair, adenylation, and adapter ligation, size-selected fragments were PCR-amplified to construct strand-specific libraries. Library quality was validated by Qubit quantification (effective concentration >2 nM) and fragment size distribution analysis. Paired-end 150 bp (PE150) sequencing was performed on the Illumina NovaSeq 6,000 platform (Biomarker Technologies, Beijing).

Raw sequencing data underwent adapter trimming and quality filtering using Trimmomatic (v0.39) to generate high-quality clean reads. Clean reads were aligned to the reference genome (*Ovis aries* ARS_UI_Ramb_v3.0) using HISAT2 (v2.2.1) with default parameters. Genomic mapping statistics—including chromosomal distribution, alignment rate, and structural annotation—were extracted from alignment files. Transcriptome assembly was performed using StringTie (v2.1.7) with reference-guided mode for downstream differential expression analysis.

### 2.7 Data processing and analysis

Statistical analysis was performed using IBM SPSS 26 software with independent samples t-test employed to analyze metabolite and gene contents (9), where results are presented as mean values and metabolites/genes with *p*-value < 0.05 were considered statistically significant; metabolites and genes showing statistically significant differences between groups were deemed differentially expressed, with fold change (FC) calculated as the ratio of mean expression values in the treatment group to those in the control group (10, 65), while variable importance in projection (VIP) scores derived from OPLS-DA models were utilized to identify differential metabolites between groups (11), retaining those with VIP > 1.0.

## 3 Results

### 3.1 Comparison of serum biochemical parameters and skin morphological indicators between Bayanbulak sheep and Turpan black sheep

**Figure 1:**
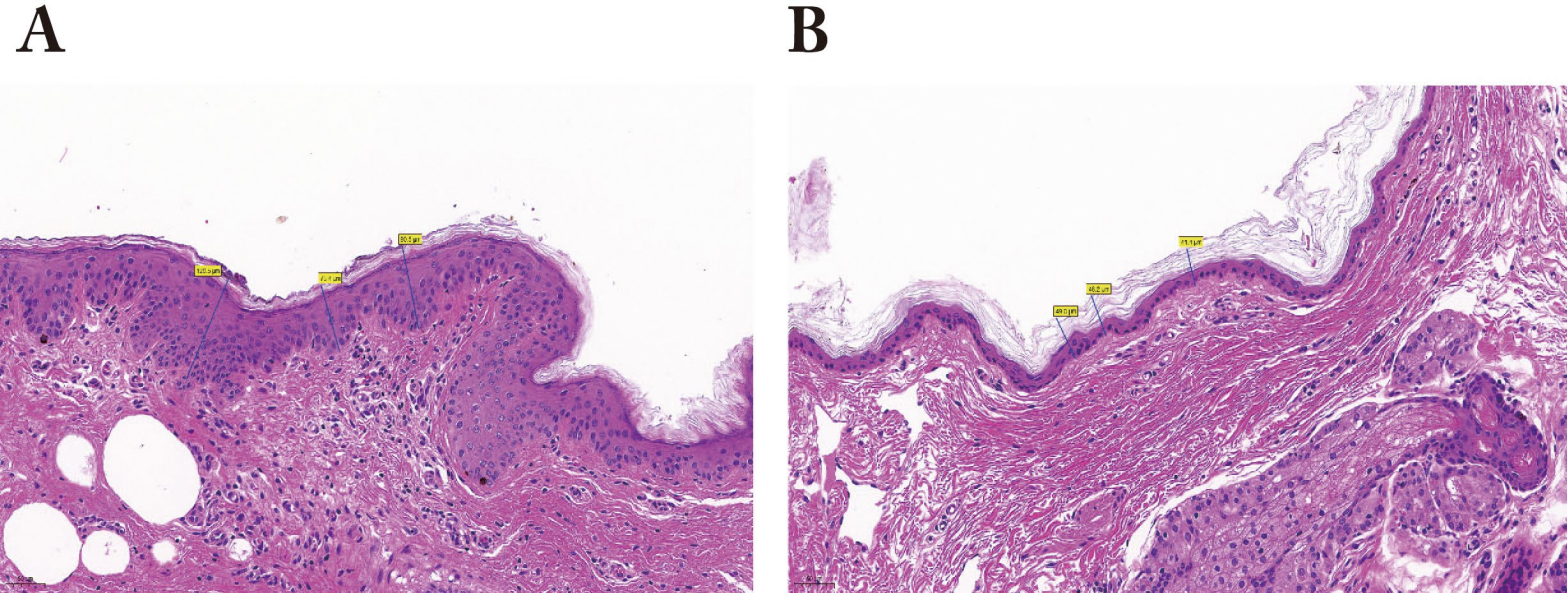
Comparative histological analysis of skin sections from Bayanbulak sheep and Turpan black sheep. **(A)** Epidermal layer of Bayanbulak sheep (20 × magnification); **(B)** Epidermal layer of Turpan black sheep (20 × magnification).

**Figure 2:**
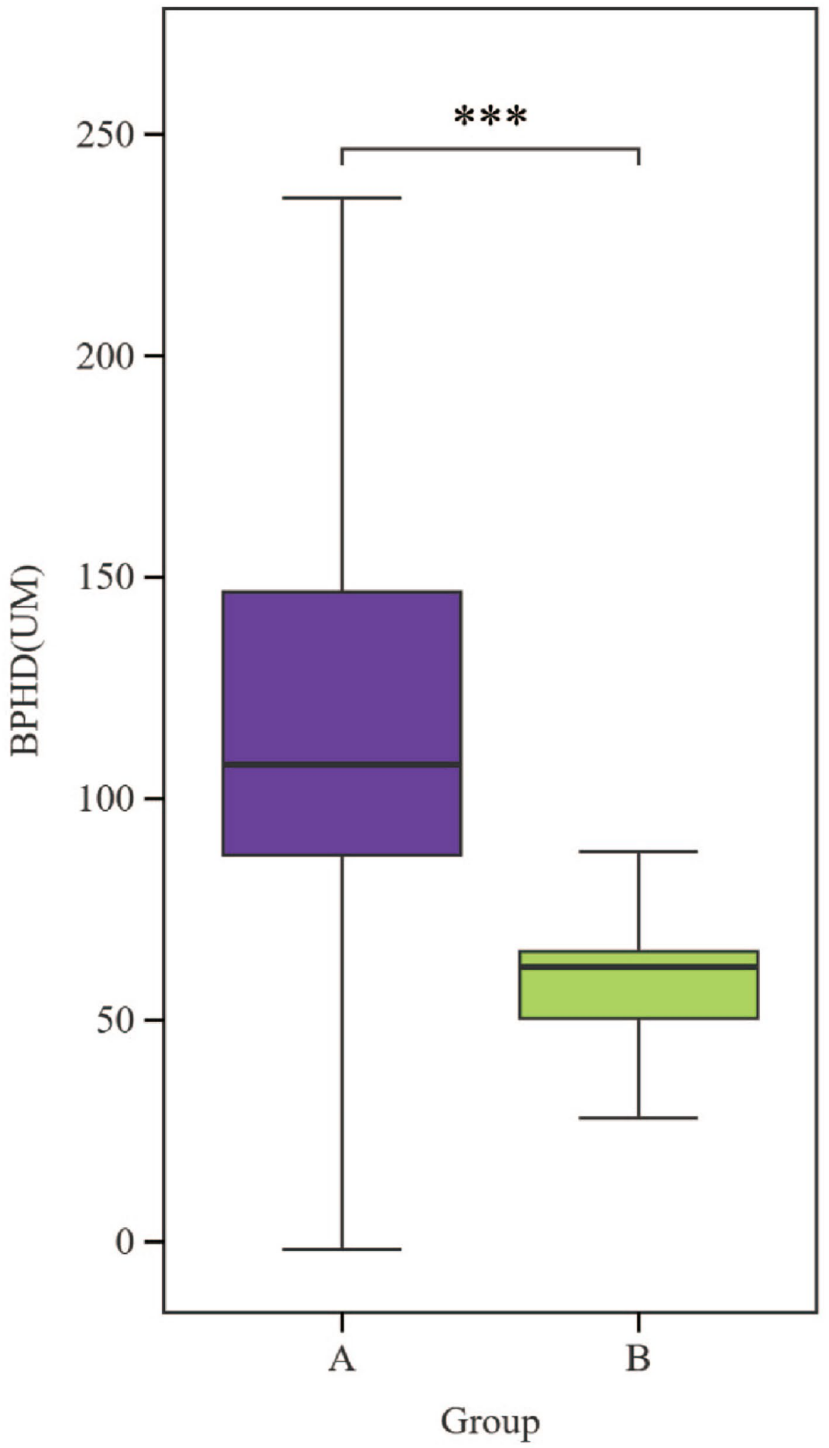
Comparative analysis of epidermal layers in skin sections from Bayanbulak sheep and Turpan black sheep. **(A)** Bayanbulak sheep (BY) **(B)** Turpan black sheep (TLF), BPDH, epidermal thickness*** denotes statistically significant differences at *p* < 0.01.

Analysis of serum biochemical parameters (Table 1) revealed significantly elevated levels in Bayanbulak sheep compared to Turpan black sheep. Five parameters showed highly significant differences (*p* < 0.01): interferon-*γ* (IFN-γ), total cholesterol (TC), glucose (GLU), total antioxidant capacity (T-AOC), and superoxide dismutase (SOD). Additionally, three parameters demonstrated significant elevations (*p* < 0.05): interleukin-2 (IL-2), triglycerides (TG), and catalase (CAT). For skin histological measurements (Table 2), epidermal thickness was found to be highly significantly greater (*p* < 0.01) in Bayanbulak sheep versus Turpan black sheep, as visually confirmed through comparative histological analysis (Figures 1, 2).

**Table 1:** Serum biochemical parameters.

Component	Abbreviation	Turpan black sheep	Bayanbulak sheep	*P*-value
Interferon-gamma	IFN-γ (ng/L)	68.50 ± 13.44	100.15 ± 16.47***	<1.0 × 10^−3^
Interleukin-2	IL-2 (ng/L)	33.59 ± 5.61	43.44 ± 8.30*	3.0 × 10^−3^
Total cholesterol	TC (mmol/L)	1.31 ± 0.24	1.72 ± 0.37***	4.0 × 10^−3^
Triglycerides	TG (mmol/L)	0.26 ± 0.11	0.36 ± 0.11*	3.0 × 10^−2^
Glucose	GLU (mmol/L)	3.56 ± 0.98	6.02 ± 1.42***	<1.0 × 10^−3^
Total antioxidant capacity	T-AOC (mmol/L)	0.71 ± 0.06	0.83 ± 0.07***	<1.0 × 10^−3^
Catalase	CAT (U/mL)	0.79 ± 0.47	1.89 ± 1.67*	4.7 × 10^−2^
Superoxide dismutase	SOD (U/mL)	92.00 ± 9.27	157.84 ± 12.35***	<1.0 × 10^−3^
Glutathione peroxidase	GSH-Px (U/mL)	38.67 ± 7.66	62.96 ± 49.88	1.1 × 10^−1^
Malondialdehyde	MDA (nmol/mL)	5.04 ± 1.62	5.59 ± 1.61	4.13 × 10^−1^

Data are expressed as mean ± SD. Significance levels: ***p < 0.01, *p < 0.05 compared to Turpan black sheep within the same parameter. Absence of symbols denotes non-significant differences (p > 0.05).

**Table 2:** Skin histological parameters.

Skin histological parameter (μm)	Turpan black sheep	Bayanbulak sheep	*P*-value
Epidermal thickness	58.97 ± 9.94	122.39 ± 14.19***	1.0 × 10^−3^

Data are expressed as mean ± SD. Significance levels: ***p < 0.01, *p < 0.05 compared to Turpan black sheep within the same parameter. Absence of symbols denotes non-significant differences (*p* > 0.05).

### 3.2 Comparative rumen metabolomics between Bayanbulak sheep and Turpan black

**Figure 3:**
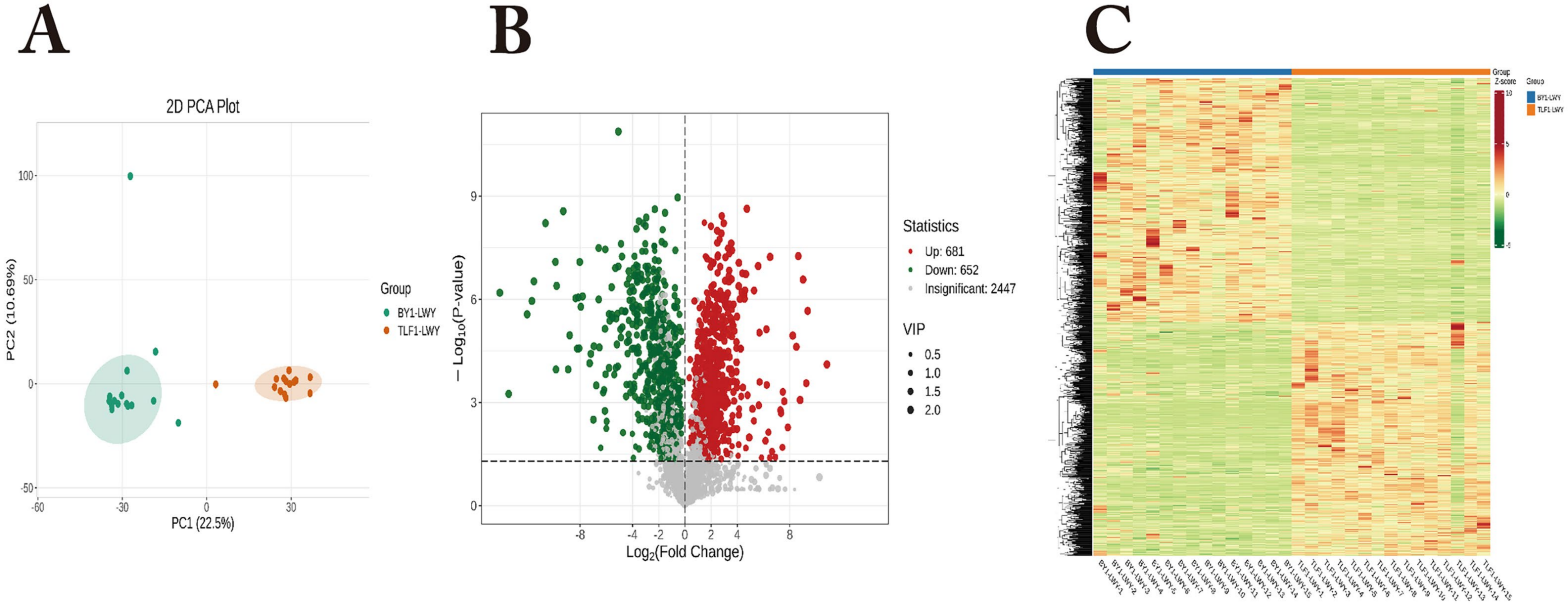
Analysis of rumen metabolite profiles in Bayanbulak sheep and Turpan black sheep. **(A)** Principal component analysis (PCA); **(B)** Quantitative statistics of differentially abundant metabolites; **(C)** Hierarchical clustering heatmap; BY1-LWY, Bayanbulak sheep rumen fluid group; TLF1-LWY, Turpan black sheep rumen fluid group.

Metabolomic profiling identified 2,447 metabolites in rumen fluid from 15 Bayanbulak sheep (high-altitude) and 15 Turpan black sheep (low-altitude). PCA and HCA (Figure 3) revealed distinct metabolic clustering between groups. Applying thresholds (|FC|>1, *p* < 0.05, VIP > 1), we detected 1,333 differentially abundant metabolites (681 up-regulated and 652 down-regulated in Bayanbulak sheep). Subsequent fold change analysis identified altitude-adaptation-associated metabolites, with adenosine monophosphate (AMP), adenosine, and ascorbic acid showing particularly elevated levels in Bayanbulak sheep.

#### 3.2.1 KEGG functional annotation of differential rumen metabolites

**Figure 4:**
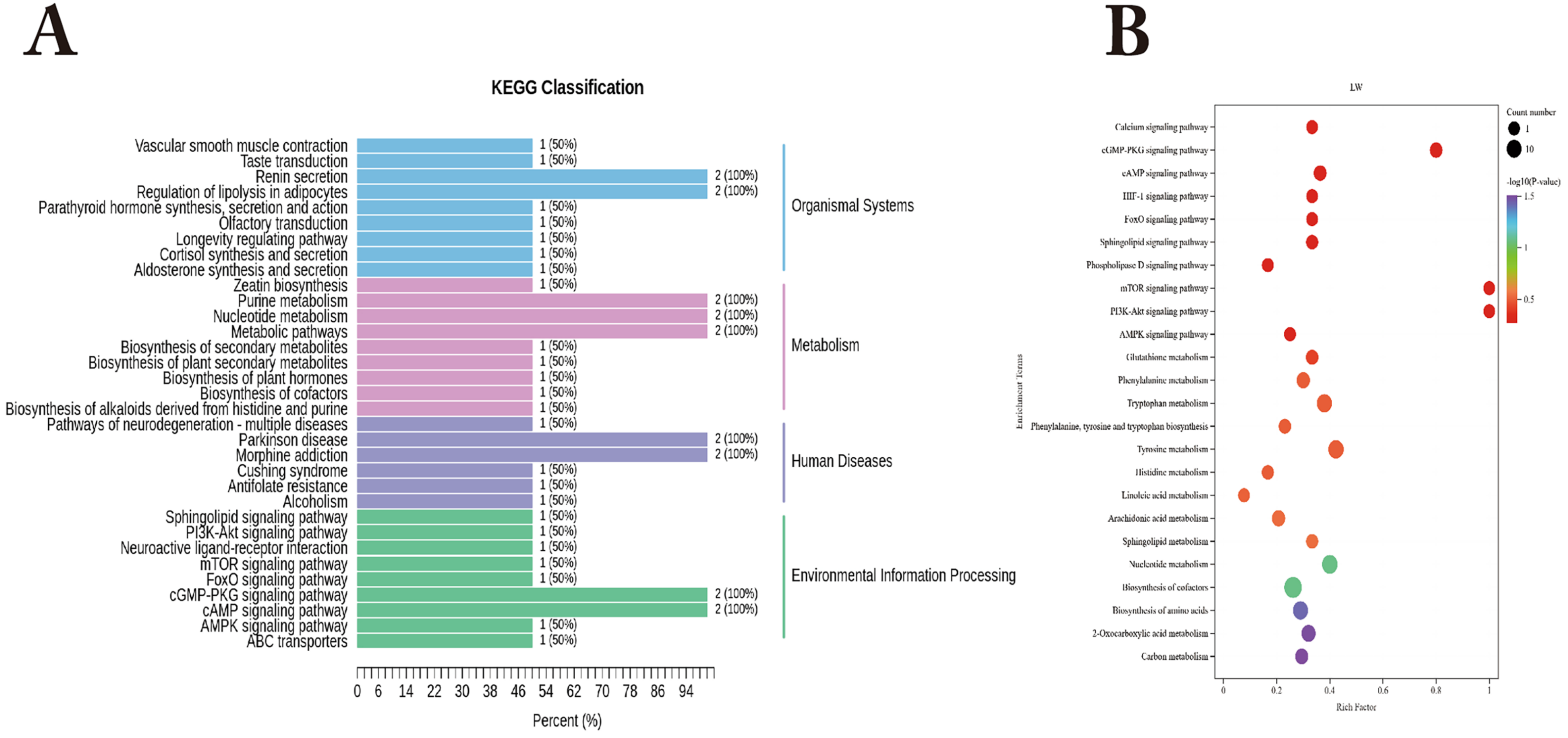
KEGG functional analysis of differential rumen metabolites in Bayanbulak sheep and Turpan black sheep. **(A)** KEGG functional classification of differential metabolites; **(B)** Pathway enrichment plot of differential metabolites.

KEGG functional annotation of differential metabolites between Bayanbulak sheep and Turpan black sheep (Figure 4) revealed predominant enrichment in the AMPK signaling pathway, mTOR signaling pathway, cAMP signaling pathway, and purine metabolism pathway, with the differential metabolite 5′-adenylic acid primarily annotated to both AMPK and mTOR signaling pathways.

### 3.3 Small intestinal metabolic profiles of Bayanbulak sheep and Turpan black sheep

**Figure 5:**
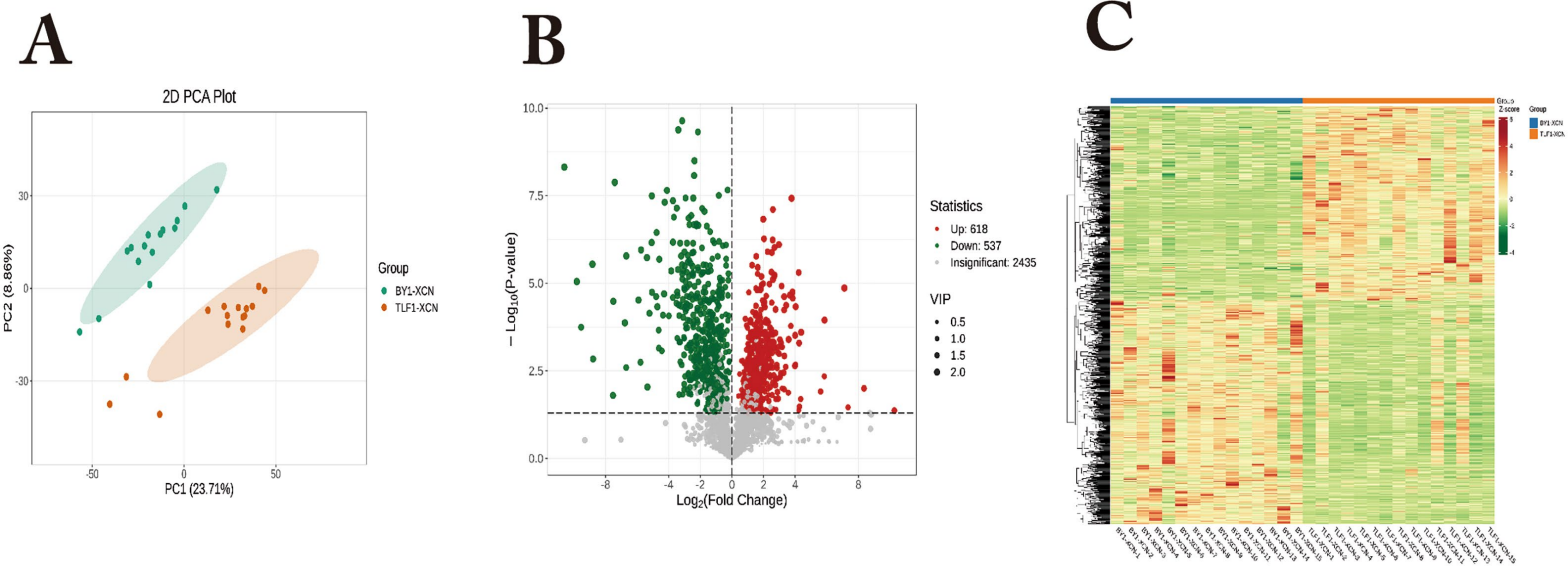
Analysis of small intestinal metabolic profiles in Bayanbulak sheep and Turpan black sheep. **(A)** Principal component analysis (PCA); **(B)** quantitative statistics of differential metabolites; **(C)** hierarchical clustering heatmap; BY1-XCN: Bayanbulak sheep small intestinal content group; TLF1-XCN, Turpan black sheep small intestinal content group.

Metabolomic profiling of small intestinal contents (15 Bayanbulak vs. 15 Turpan black sheep) identified 2,435 metabolites. PCA and hierarchical clustering (Figure 5) showed distinct metabolic separation between altitude groups. Using |FC|>1, *p* < 0.05 and VIP > 1 thresholds, we found 1,155 differentially abundant metabolites (618 up- and 537 down-regulated in Bayanbulak sheep). Key adaptation-related changes included elevated spermidine, L-glutamate and oleic acid, but reduced nicotine metabolites in Bayanbulak sheep.

#### 3.3.1 KEGG functional annotation of differential small intestinal metabolites

**Figure 6:**
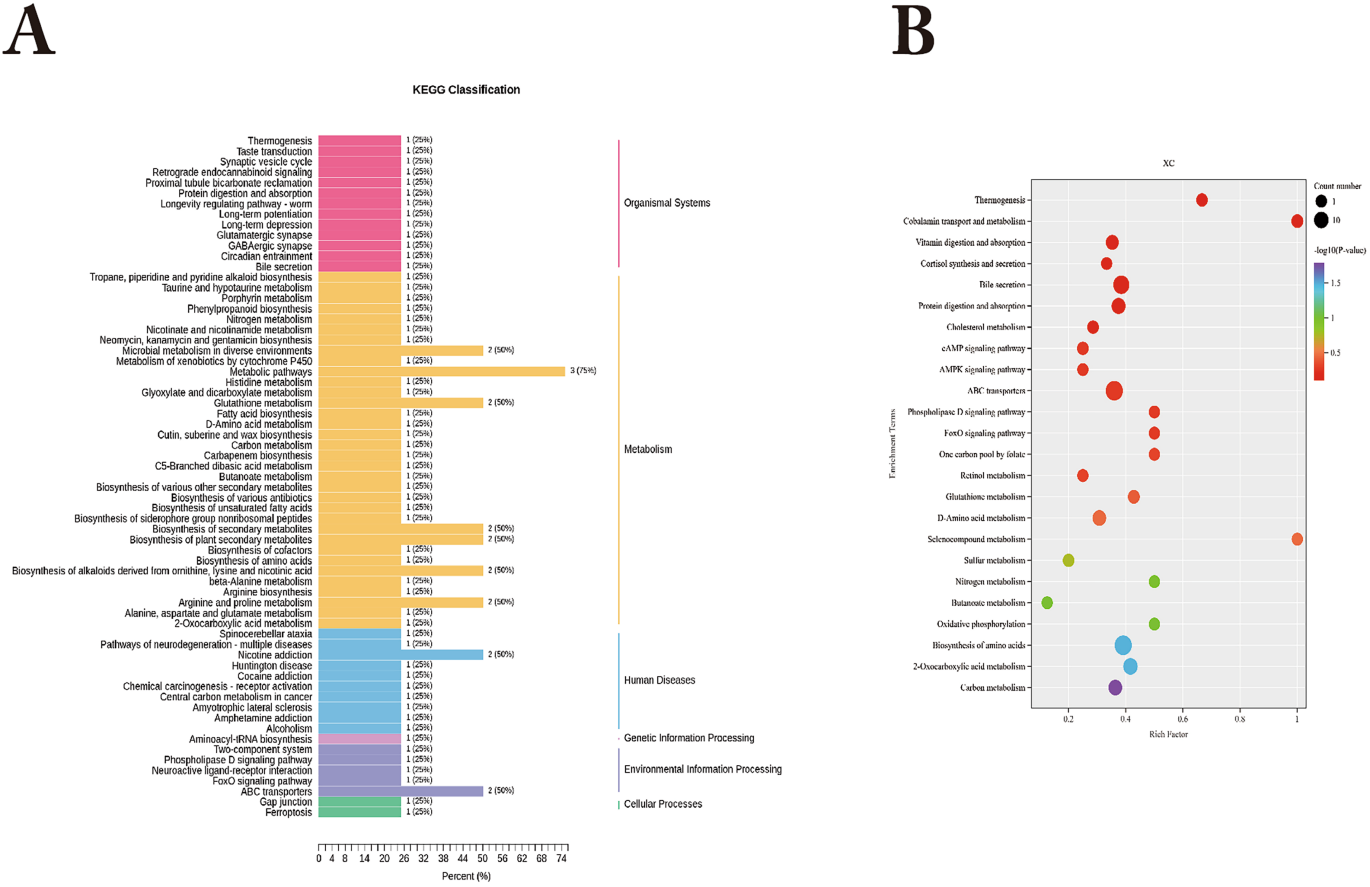
KEGG functional analysis of differential metabolites in small intestine of Bayanbulak sheep and Turpan black sheep. **(A)** KEGG functional classification of differential metabolites; **(B)** Pathway enrichment plot of differential metabolites.

KEGG functional annotation of differential metabolites between Bayanbulak sheep and Turpan black sheep (Figure 6) revealed predominant enrichment in glutathione metabolism, thermogenesis pathway, ferroptosis, and fatty acid biosynthesis pathways, with the differential metabolites spermidine and L-glutamate primarily annotated to both glutathione metabolism and ferroptosis pathways.

### 3.4 Differential fecal metabolic profiles of Bayanbulak sheep and Turpan black sheep

**Figure 7:**
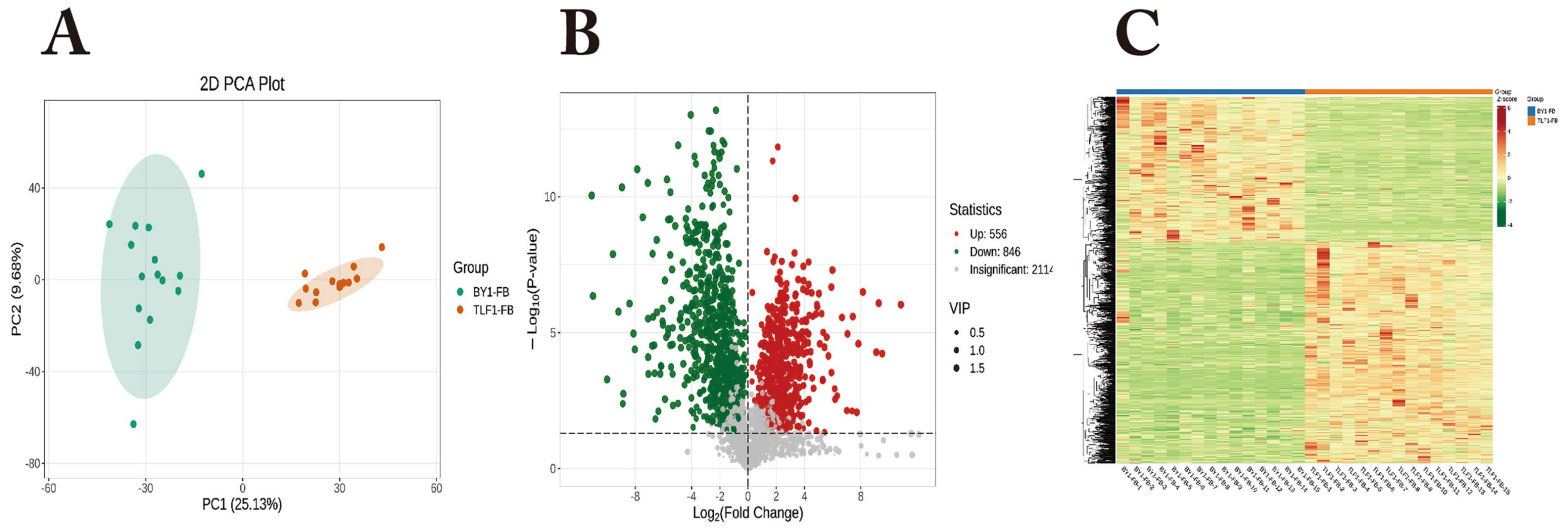
Analysis of fecal metabolic profiles in Bayanbulak sheep and Turpan black sheep. **(A)** Principal component analysis (PCA); **(B)** quantitative statistics of differential metabolites; **(C)** hierarchical clustering heatmap; BY1-FB, Bayanbulak sheep fecal group; TLF1-FB, Turpan black sheep fecal group.

A total of 2,114 metabolites were identified in fecal samples from 15 Bayanbulak sheep and 15 Turpan black sheep. Principal component analysis (PCA) and hierarchical clustering analysis (Figure 7) revealed distinct clustering of fecal metabolites between altitudinal groups. Using thresholds of fold change|FC|>1, *p* < 0.05, and variable importance in projection (VIP) > 1, we identified 1,402 differentially abundant metabolites between breeds. Among these, 556 metabolites were up-regulated and 846 were down-regulated in Bayanbulak sheep. Further screening for high-altitude adaptation relevance demonstrated significantly elevated concentrations of allantoin metabolites in the Bayanbulak group compared to the Turpan black sheep group.

#### 3.4.1 KEGG functional annotation of differential fecal metabolites

**Figure 8:**
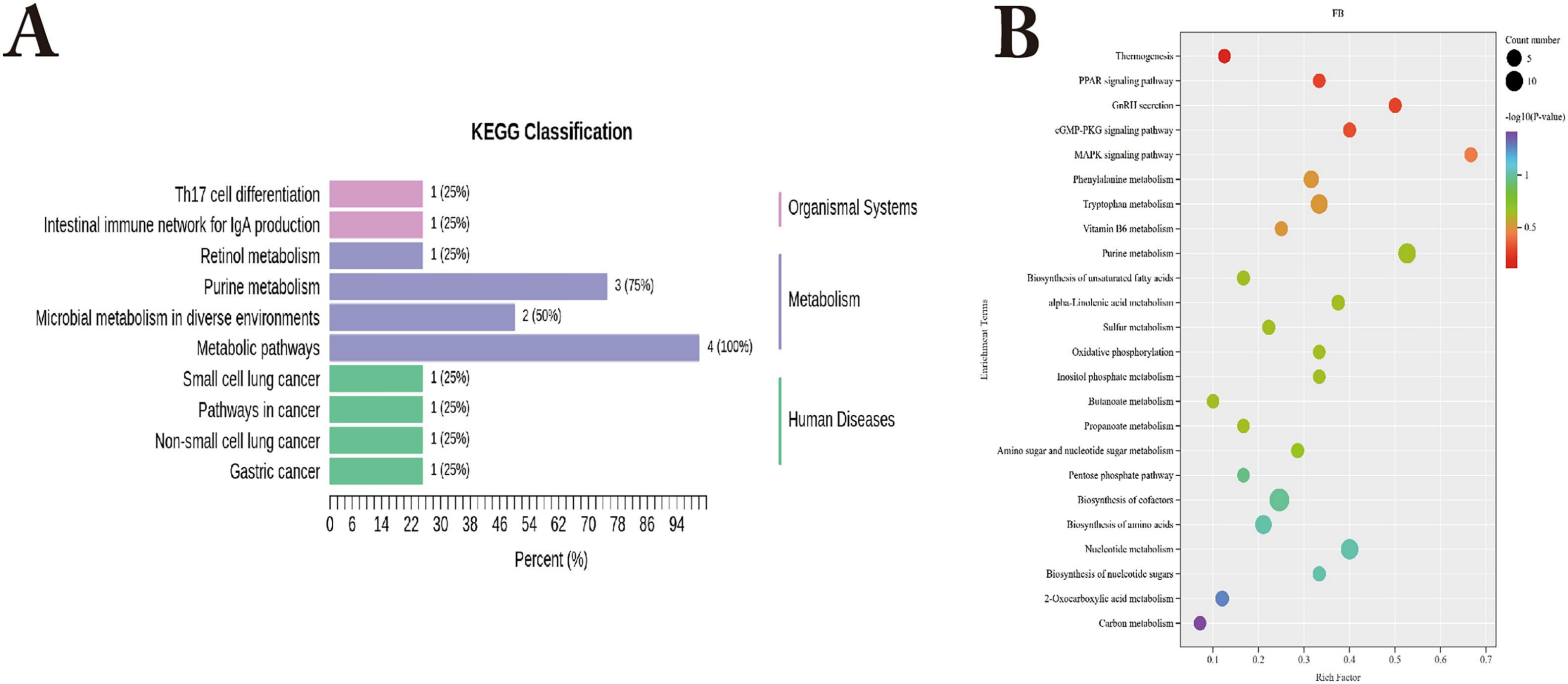
KEGG functional analysis of differential metabolites in small intestine of Bayanbulak sheep and Turpan black sheep. **(A)** KEGG functional classification of differential metabolites; **(B)** Pathway enrichment plot of differential metabolites.

KEGG functional annotation of differential metabolites between Bayanbulak sheep and Turpan black sheep (Figure 8) revealed predominant enrichment in purine metabolism, Th17 cell differentiation, and intestinal IgA production immune network pathways, with the differential metabolite allantoin primarily annotated to purine metabolism.

### 3.5 Comparative analysis of digestive tract metabolites between Bayanbulak and Turpan black sheep

**Figure 9:**
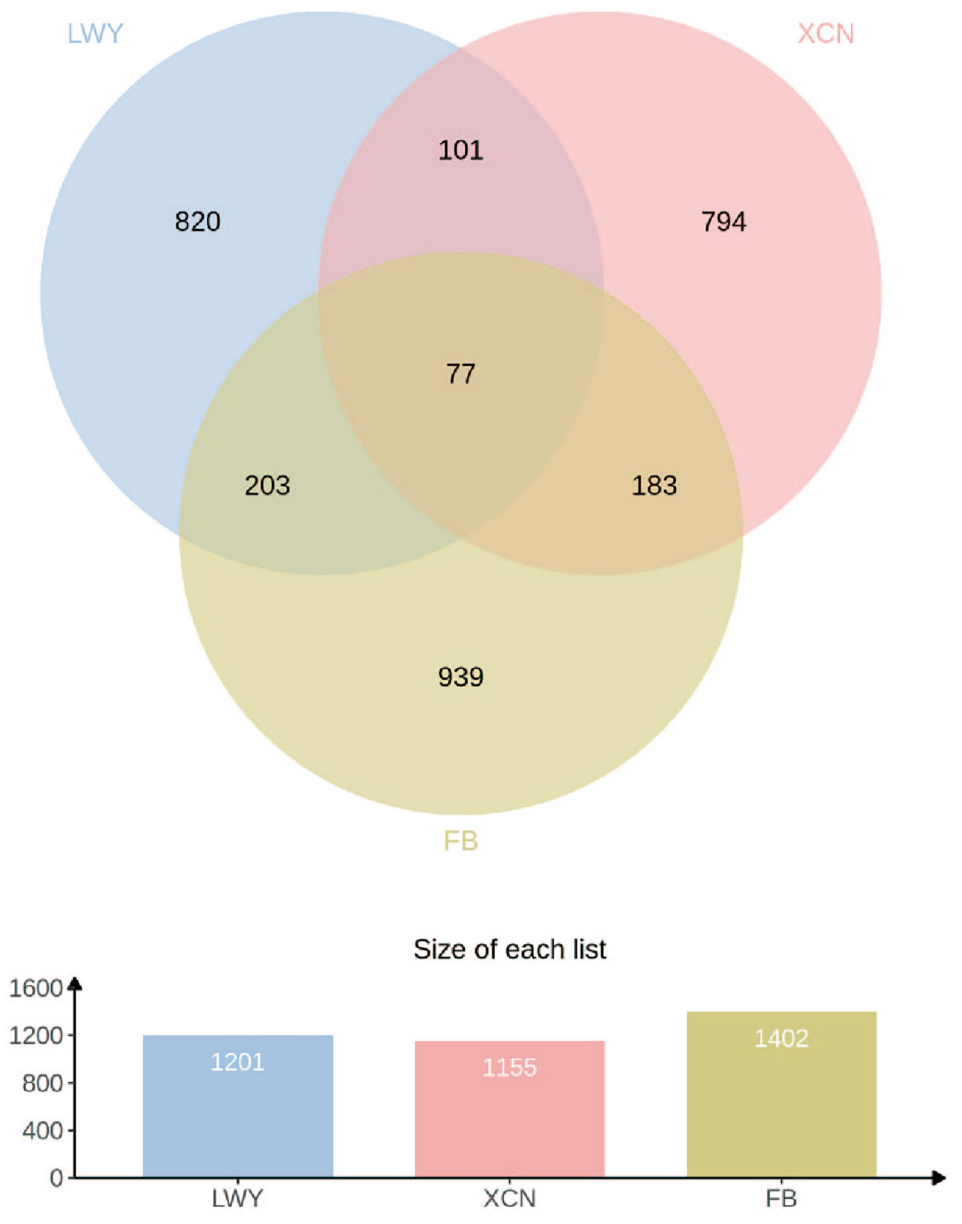
Analysis of shared metabolites in rumen fluid, small intestinal content, and feces of Bayanbulak sheep and Turpan black sheep. LWY, rumen fluid; XCN, small intestinal content; FP, feces.

Comparative analysis of ruminal, small intestinal, and fecal metabolites between Bayanbulak and Turpan black sheep revealed 77 shared metabolites (Figure 9). Notably, 4-hydroxy-3-methoxybenzaldehyde exhibited compartment-specific regulation: significantly up-regulated in ruminal metabolites but down-regulated in both small intestinal and fecal metabolites of Bayanbulak sheep versus Turpan black sheep (Table 3). In contrast, pinacidil demonstrated consistent up-regulation across all three digestive compartments, whereas 4′-hydroxychalcone showed pan-enteric down-regulation. Vanillin, pinacidil, and 4′-hydroxychalcone exhibited significant alterations across all three digestive compartments in Bayanbulak sheep, and were associated with high-altitude adaptation mechanisms including antioxidant defense, vasodilation, and oxidative stress regulation.

**Table 3:** Comparative analysis of differential digestive tract metabolites between Bayanbulak and Turpan black sheep.

Compound	Compartment	*P*-value	Regulation
Vanillin	Rumen	2.786E-04	Up
	Small intestine	5.793E-03	Down
	Feces	1.066E-03	Down
Pinacidil	Rumen	2.885E-05	Up
	Small intestine	1.440E-03	Up
	Feces	1.905E-04	Up
4’-Hydroxychalcone	Rumen	2.185E-12	Down
	Small intestine	2.185E-12	Down
	Feces	2.185E-12	Down

Statistically significant differences (*P* < 0.05) indicate differential regulation for the specified metabolite within a given digestive compartment. Non-significant differences (*p* > 0.05) denote absence of measurable regulation.

### 3.6 Differential gene expression analysis between Bayanbulak and Turpan black sheep

**Figure 10:**
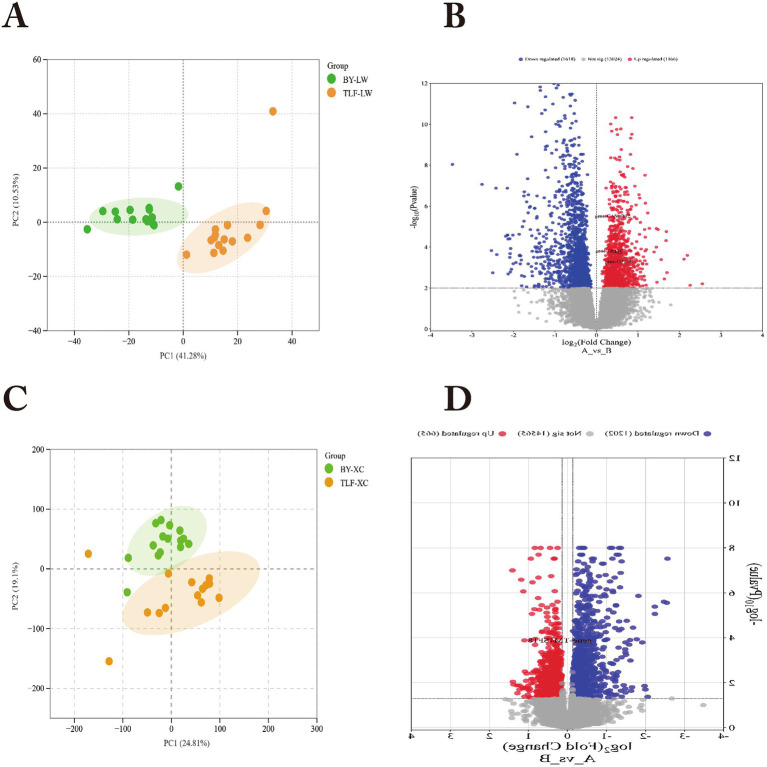
Multi-tissue transcriptomic profiling of differentially expressed genes in rumen and small intestine of Bayanbulak vs. Turpan black sheep. **(A)** Principal component analysis (PCA); **(B)** Differential metabolite quantification; BY, Bayanbulak sheep group; TLF, Turpan black sheep group; LW, rumen; XC, small intestine **(C)** Principal component analysis (PCA); **(D)** Differential metabolite quantification.

Gene expression analysis of ruminal tissues (15 Bayanbulak vs. 15 Turpan black sheep) identified 13,024 genes. PCA showed clear altitude-dependent separation (Figure 10A). Stringent analysis (*p* < 0.01) revealed 2,784 DEGs (1,166 up-, 1,618 downregulated; Figure 10B), including upregulated cold-adaptation genes CPT1B, CPT1C and SLC7A2 in Bayanbulak sheep. Small intestinal analysis detected 15,407 genes with distinct altitude clustering (Figure 10C). We identified 1,025 DEGs at *p* < 0.01 (332 up-, 693 downregulated; Figure 10D), notably downregulated TM4SF18 in Bayanbulak sheep.

#### 3.6.1 Comparative analysis of rumen and intestinal metabolome and gene expression differences between Bayanbulak sheep and Tulfan black sheep

**Figure 11:**
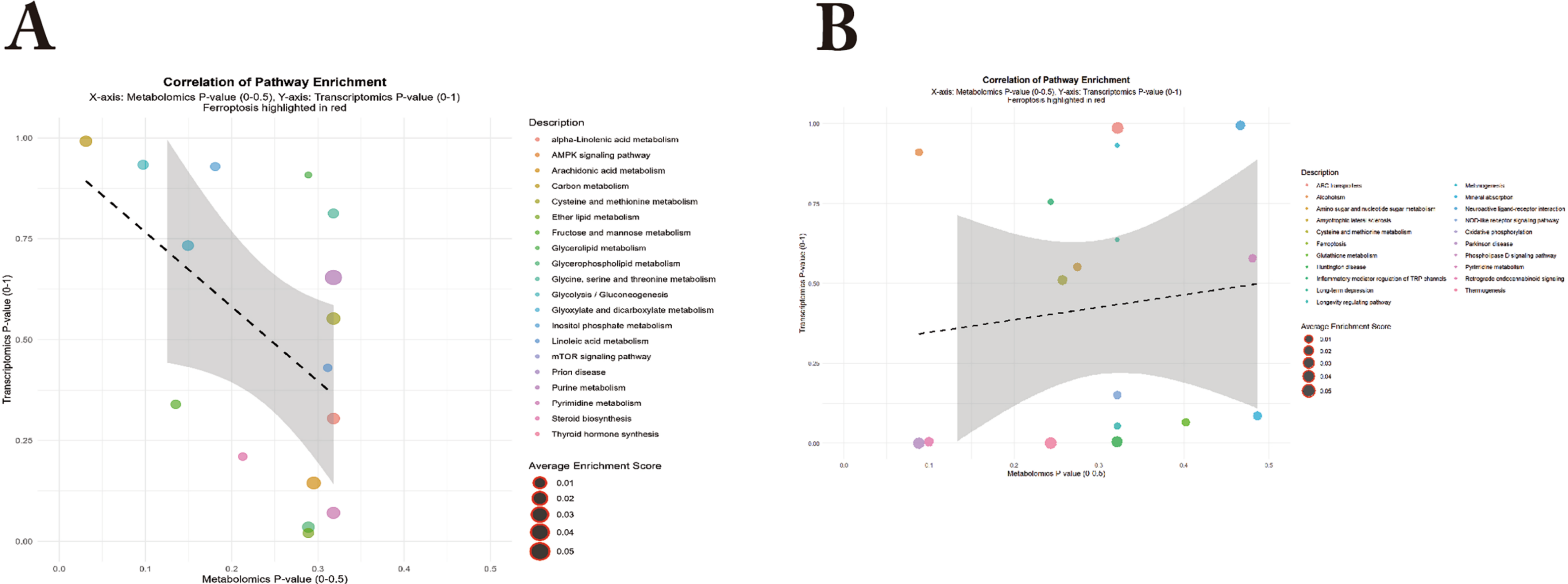
Integrated co-enrichment pathway analysis of rumen and small intestine in Bayanbulak sheep and Turpan black sheep. **(A)**. Bubble plot of co-enriched pathways in rumen; **(B)**. Bubble plot of co-enriched pathways in small intestine.

The comparison of rumen and small intestine metabolic and gene differences between Bayanbulak sheep and Turpan black sheep, as shown in the provided text, reveals significant differences in metabolic pathways and gene expression. Specifically, for the rumen, transcriptome and metabolome co-analysis, as depicted in Figure 11A, indicates that KEGG enrichment analysis of differentially expressed genes (DEGs) and differential metabolites (DMS) show enrichment in pathways such as the AMPK signaling pathway and mTOR signaling pathway. Compared to the Turpan black sheep group, the Bayanbulak sheep group exhibited significantly higher levels of 5′-adenosine monophosphate, and the expression of genes such as CPT1B, CPT1C, and CASTOR2 was significantly upregulated. For the small intestine, transcriptome and metabolome co-analysis, as shown in Figure 11B, revealed that KEGG enrichment analysis of DEGs and DMS showed enrichment in the ferroptosis pathway. Compared to the Turpan black sheep group, the Bayanbulak sheep group showed significantly higher levels of L-glutamine, and the expression of the TM4SF18 gene was significantly downregulated.

## 4 Discussion

### 4.1 Analysis of serum biochemical parameters and skin morphological indicators in Bayanbulak sheep versus Turpan black sheep

To elucidate the comprehensive adaptation mechanisms of animals to high-altitude environments, this study systematically examined the coordinated adaptation strategies of Bayanbulak sheep under high-altitude hypoxic and low-temperature conditions through integrated analysis of serum biochemical parameters and skin histomorphometric data across four physiological dimensions: immunomodulation, energy metabolism, antioxidant defense, and physical barrier function.

In the immunological dimension, Bayanbulak sheep exhibited significant elevations in serum interferon-*γ* (IFN-γ) and interleukin-2 (IL-2), indicating enhanced immune system activation that corresponds with established immune adaptation patterns in high-altitude mammals including Tibetan sheep (12, 66). Under the combined stressors of hypoxia and cold exposure, these animals demonstrate upregulated IFN-γ expression, which mediates cellular functions including proliferation inhibition, apoptosis promotion, and immune response activation for targeted elimination of transformed cells (13). The synchronized increase of both IFN-γ and IL-2 not only reflects potentiated Th1-type immune responses but also suggests optimized immune resource allocation under environmental duress through regulatory T-cell homeostasis adjustments (14).

Metabolically, Bayanbulak sheep displayed comprehensive elevation in serum total cholesterol (TC), triglycerides (TG), and glucose (GLU) levels, revealing substantial remodeling of energy storage and utilization pathways. Compared to their low-altitude Turpan black sheep counterparts, Bayanbulak sheep demonstrated enhanced lipid mobilization and glucose metabolic capacity, consistent with documented metabolic adaptations in Mongolian sheep that augment fat metabolism for thermal regulation in cold environments (6). Cholesterol serves dual roles as both a structural component of cell membranes and a precursor for steroid hormone synthesis, while its metabolic derivatives—particularly bile acids—facilitate lipid digestion and absorption (15, 16), suggesting that Bayanbulak sheep potentially improve dietary energy harvest efficiency through these mechanisms. Furthermore, triglycerides function as deposited energy reserves (17) to support cold adaptation, while glucose operates not only as an immediate energy substrate but also sustains reduced glutathione (GSH) regeneration via the pentose phosphate pathway (18), thereby exhibiting dual functionality in both energy provision and antioxidant defense.

Regarding antioxidant systems, Bayanbulak sheep manifested significantly enhanced activities of total antioxidant capacity (T-AOC), superoxide dismutase (SOD), and catalase (CAT), constituting an integrated enzymatic defense network where SOD catalyzes the conversion of superoxide anions to hydrogen peroxide (19) and CAT subsequently decomposes hydrogen peroxide into water and molecular oxygen (20), effectively disrupting reactive oxygen species cascade reactions. This antioxidant coordination aligns with oxidative stress adaptation strategies documented in high-altitude species such as yaks (21, 67), reflecting an evolutionarily conserved pathway for maintaining redox equilibrium under hypoxic conditions.

The investigation additionally identified significantly greater epidermal thickness in Bayanbulak sheep compared to Turpan black sheep, suggesting this morphological adaptation provides augmented physical protection against high-altitude environmental challenges including intense ultraviolet radiation and low-temperature exposure. This observation correlates with established understanding of epidermal stratum corneum function as the outermost protective layer that resists diverse environmental physicochemical stressors (22).

In synthesis, the high-altitude adaptation of Bayanbulak sheep represents a multidimensional integrative process rather than reliance on isolated mechanisms, achieved through synergistic coordination across immune system activation, metabolic reprogramming, antioxidant enhancement, and structural barrier reinforcement. These findings substantially advance comprehension of animal adaptation to high-altitude environments and establish theoretical foundations for future initiatives in livestock genetic improvement and environmental adaptation breeding. The differential metabolites vanillin, pinacidil, and 4′-hydroxychalcone identified across three distinct digestive compartments further demonstrate associations with high-altitude adaptation mechanisms encompassing antioxidant defense, vasodilation, and oxidative stress regulation.

### 4.2 Metabolomics of Bayanbulak sheep and Turpan black sheep

#### 4.2.1 Rumen metabolomic analysis of Bayanbulak sheep and Turpan black sheep

Through comparative analysis of the rumen metabolome between Bayanbulak sheep and Turpan black sheep, this study reveals a crucial adaptive dimension in energy metabolism and oxidative balance regulation in high-altitude mammals: orchestrating energy supply, thermoregulation, and antioxidant defense through ruminal metabolic adaptations to counter persistent hypoxic stress.

Hypoxic conditions restrict mitochondrial oxidative phosphorylation, diminishing ATP synthesis while upregulating AMP, which subsequently activates the AMPK pathway by binding to the cystathionine *β*-synthase (CBS) domain of its *γ* subunit (23), mirroring mechanisms observed in other high-altitude ruminants like Tibetan sheep (24, 68). This activation enhances glucose uptake via upregulated GLUT4 expression and GLUT1 membrane translocation to bypass insulin resistance, while simultaneously reprogramming lipid metabolism through ACC1/ACC2 inhibition to drive fatty acid β-oxidation (25–27), thereby prioritizing energy allocation to critical organs including the heart and brain. Concurrently, AMPK-mediated suppression of mTORC1 complex activity (28) reduces energy expenditure on mitochondrial respiration and ribosome biogenesis, aligning with fat mobilization strategies during winter forage scarcity—a metabolic pattern similarly observed in Mongolian sheep during cold adaptation (6). This mechanism becomes further reinforced under low-temperature conditions through preferential lipid over glycogen catabolism, simultaneously lowering oxygen demand and extending energy availability.

Intraruminal 5′-AMP undergoes conversion to adenosine via 5′-nucleotidase, activating central adenosine A1 receptors (29) to suppress hypothalamic thermoregulatory centers. This mechanism shows remarkable consistency with findings from murine models (30, 31) where exogenous AMP induced hypometabolic states to alleviate hypoxic injury, significantly reducing body heat loss in cold environments. Furthermore, adenosine-mediated suppression of thermogenesis and promotion of feeding behavior through central A1 receptor activation resonates with avian and mammalian studies (32–34), reflecting an evolutionarily conserved “energy conservation priority” strategy in resource-limited environments.

Notably, 5′-AMP enhances superoxide dismutase (SOD), catalase (CAT), and glutathione peroxidase (GSH-Px) activities while increasing reduced glutathione (GSH) content and decreasing malondialdehyde (MDA) levels. This antioxidant mechanism parallels observations in murine models (35), effectively neutralizing mitochondrial ROS bursts induced by hypoxia and synergistic damage from UV radiation.

In summary, Bayanbulak sheep maintain energy homeostasis amidst forage scarcity and extreme climates through integrated thermoregulation, feeding promotion, and energy conservation mechanisms, thereby significantly enhancing population fitness and survival competitiveness.

#### 4.2.2 Small intestinal metabolomic analysis of Bayanbulak sheep and Turpan black sheep

Through comparative analysis of the small intestinal metabolome between Bayanbulak sheep and Turpan black sheep, this study reveals another crucial adaptive strategy in nutrient utilization and oxidative homeostasis regulation in high-altitude ruminants at the critical interface of digestion and absorption—the small intestinal level. The findings demonstrate that Bayanbulak sheep establish a comprehensive metabolic adaptation aimed at enhancing intestinal antioxidant capacity, promoting energy storage, and reducing unnecessary energy consumption through upregulation of spermidine, L-glutamate, and oleic acid, coupled with downregulation of nicotine.

Consistent with the systemic antioxidant enhancement observed in rumen and serum, the significant enrichment of spermidine and L-glutamate in glutathione metabolism pathways within the small intestine—with L-glutamate additionally enriched in ferroptosis pathways—holds particular physiological significance given the small intestine’s crucial role in nutrient absorption and transformation. Spermidine significantly enhances the clearance capacity for lipid peroxides and superoxide anions by elevating glutathione peroxidase (GSH-Px) and superoxide dismutase (SOD) activities (19, 35, 36). This mechanism is not isolated, as related studies have observed positive correlations between polyamine metabolite accumulation and antioxidant enzyme activities in tissues (37, 69), suggesting that enhanced intestinal antioxidant defense through polyamine compounds may represent a convergent adaptation strategy among high-altitude ruminants. More importantly, this study further reveals that L-glutamate not only serves as the core precursor for glutathione synthesis, maintaining continuous synthesis of intracellular reduced glutathione (GSH) and redox homeostasis (38), but also functions as an important oxidative fuel for enterocytes, with its upregulation potentially optimizing overall nutrient utilization efficiency indirectly. Research indicates that L-glutamate supplementation increases key intermediate metabolites such as glutamine and *α*-ketoglutarate in jejunal mucosa and enhances plasma essential amino acid levels in weaned piglets (39–41). Accordingly, it is hypothesized that L-glutamate enrichment in Bayanbulak sheep likely enhances intestinal integrity and promotes nutrient digestion and absorption through similar mechanisms, thereby maximizing energy and nutrient acquisition under limited foraging conditions—a sophisticated adaptation to the ecological pressure of seasonal forage scarcity commonly associated with high-altitude environments.

Oleic acid, as a product of fatty acid biosynthesis pathways, serves as a preferred substrate for acyl-CoA cholesterol acyltransferase (ACAT) and diacylglycerol acyltransferase (DGAT), enzymes responsible for cholesterol ester and triglyceride synthesis, respectively (42). The increased oleic acid content enhances cholesterol ester and triglyceride synthesis. Triglycerides represent important metabolic fuels that can supply energy-demanding tissues during energy deficiency or replenish white adipose tissue (WAT) for storage during energy surplus (43). This finding corroborates our observed elevation of serum total cholesterol and triglyceride levels, indicating a preference for energy storage as fat when possible, to cope with energy crises caused by winter forage scarcity. This strategy aligns with the adaptation mechanism observed in Mongolian sheep, which increase fat deposition to resist severe cold (6).

The downregulation of nicotine in thermogenesis pathways inhibits the activation of the sympathetic nervous system and brown adipose tissue (BAT)-mediated thermogenesis (44), enabling Bayanbulak sheep to effectively reduce basal metabolic rate (BMR), thereby decreasing oxygen demand—a crucial adaptation to hypoxic environments. By suppressing energy expenditure related to thermogenesis while enhancing feeding behavior to compensate for energy reserves (45), nicotine downregulation may synergize with AgRP-mediated antagonism of *α*-MSH to promote feeding behavior and reduce energy consumption.

#### 4.2.3 Fecal Metabolomic analysis of Bayanbulak sheep and Turpan black sheep

Through comparative analysis of fecal metabolites between Bayanbulak sheep and Turpan black sheep, this study reveals novel dimensions of high-altitude adaptation in systemic antioxidant regulation and nitrogen metabolic balance from the terminal phase of organismal metabolism. The findings demonstrate significant upregulation of allantoin—a key intermediate in purine metabolism—in Bayanbulak sheep, suggesting its potential crucial role in systemic antioxidant defense and metabolic recycling.

Echoing the glutathione-centered antioxidant system observed in rumen and small intestine, the accumulation of allantoin in feces indicates that Bayanbulak sheep establish a cross-compartment, multi-molecule coordinated antioxidant network. In plant research, allantoin has been identified as a key signaling molecule responding to oxidative stress, with its accumulation level positively correlated with organismal stress tolerance (46). More importantly, allantoin possesses direct reactive oxygen species (ROS) scavenging capacity (47), a mechanism likely functional in Bayanbulak sheep, assisting in clearing excess ROS generated by high-altitude hypoxic environments and consequently reducing production of lipid peroxidation products like MDA. This finding corroborates our observed enhancements in serum SOD and CAT activities alongside relatively stable MDA levels, collectively indicating allantoin’s integral role in maintaining systemic redox homeostasis in Bayanbulak sheep.

Notably, the protective effects of allantoin are not isolated but reinforced through synergistic interactions with polyamine metabolism. Studies demonstrate that allantoin promotes the accumulation of putrescine, spermine, and spermidine (48, 49), providing strong mutual validation with the observed spermidine upregulation in the small intestine from this study, revealing coordinated mechanisms between purine metabolism and polyamine metabolism across different tissues and organs. The upregulated spermidine can further activate glutathione peroxidase and superoxide dismutase (19), thereby establishing a multi-layered, amplified antioxidant defense system comprising signaling molecules (allantoin) - effector molecules (polyamines) - antioxidant enzymes (GSH-Px, SOD) building upon allantoin’s direct ROS scavenging capacity.

In summary, the upregulation of allantoin in fecal metabolites of Bayanbulak sheep not only represents the terminal manifestation of their response to high-altitude oxidative stress but also, through cross-compartment synergy with the polyamine system, closely links purine metabolism with the organism’s overall antioxidant defense network. This discovery retrospectively interprets physiological significance from the metabolic endpoint, providing terminal evidence for comprehensively understanding the systemic metabolic remodeling in ruminant adaptation to high-altitude environments.

### 4.3 Comparative analysis of gastrointestinal tract metabolites in Bayanbulak sheep and Turpan black sheep

Through integrated analysis of the metabolomes from three consecutive digestive tract segments (rumen, small intestine, and feces) of Bayanbulak sheep and Turpan black sheep, this study reveals the multi-level, cross-compartment metabolic协同 adaptation mechanisms achieved by high-altitude ruminants within their digestive systems. Among the 77 identified common differential metabolites, vanillin, pinacidil, and 4′-hydroxychalcone exhibited unique abundance variation patterns across the three digestive segments, collectively forming a comprehensive metabolic adaptation network ranging from local microenvironment regulation to systemic physiological adaptation.

The upregulation of vanillin in the rumen contrasted with its downregulation in the small intestine and feces reflects compartmentalized utilization of its bioactivity during digestion. As the fermentation center, ruminal upregulation of vanillin may maintain ruminal microenvironment homeostasis through its antioxidant properties (50) and protective effects (51), while simultaneously enhancing host energy acquisition by increasing volatile fatty acid concentration (50, 52), consistent with the strategy of high-altitude ruminants like Tibetan sheep optimizing rumen fermentation to improve energy efficiency (53). Its downregulation in the small intestine, considering vanillin’s capacity to reduce physical and adipose tissue burden (52), may represent metabolic programming that reduces lipolysis and promotes energy storage for cold adaptation, reflecting differential utilization of the same metabolite’s functions across digestive phases.

In contrast, the consistent upregulation of pinacidil across all three segments suggests its potential absorption into the circulatory system for systemic physiological functions. As a potassium channel opener, pinacidil induces vasodilation and increases local blood flow (51). Under high-altitude hypoxic conditions, this mechanism potentially improves blood perfusion and oxygen supply to intestinal and peripheral tissues, functionally converging with enhanced vasodilatory responses observed in high-altitude adapted human populations (54). The persistent upregulation of pinacidil highlights Bayanbulak sheep’s unique adaptation strategy of regulating metabolites to improve tissue oxygen supply.

The consistent downregulation of 4′-hydroxychalcone conversely demonstrates Bayanbulak sheep’s precise avoidance of oxidative stress. This compound has been confirmed to deplete glutathione, increase reactive oxygen species, and impair mitochondrial function (52). Its widespread downregulation throughout the digestive tract can be viewed as an active regulatory measure taken by the organism to reduce endogenous oxidative damage sources and maintain systemic redox balance. This complements the robust antioxidant capacity we observed in serum and intestinal tissues, collectively forming a dual defense strategy of “enhancing protection” while “reducing harm.”

In summary, connections exist among ruminal metabolites, small intestinal metabolites, and fecal metabolites, and their combined analysis reveals these metabolites’ significant roles in Bayanbulak sheep’s adaptation to high-altitude cold environments.

### 4.4 Integrated analysis of rumen and small intestinal metabolome and transcriptome in Bayanbulak sheep and Turpan black sheep

This study aims to investigate the high-altitude adaptation mechanisms of Bayanbulak sheep through integrated analysis of rumen and small intestinal metabolomes and transcriptomes compared with low-altitude Turpan black sheep. The findings demonstrate upregulated expression of CPT1B, CPT1C, and CASTOR2 genes in the rumen of Bayanbulak sheep, alongside downregulated expression of TM4SF18 gene in their small intestine.

In core energy metabolism pathways, the coordinated upregulation of CPT1C and CPT1B in rumen tissue highlights the central role of fatty acid oxidation in high-altitude energy supply. As the rate-limiting enzyme for fatty acid *β*-oxidation (55), CPT1 family member CPT1C is specifically induced under metabolic stress conditions such as hypoxia (56). In this study, the high-altitude hypoxic environment potentially activates the AMPK-p53 signaling axis (57, 58), driving transcriptional upregulation of CPT1C. This mechanism aligns with findings from (59), where CPT1C enhances fatty acid uptake, promotes intracellular ATP generation, and maintains redox homeostasis. Notably, CPT1B upregulation not only facilitates fatty acid oxidation but also promotes adipogenesis when overexpressed in preadipocytes (60). This indicates dual purposes in lipid metabolism regulation in Bayanbulak sheep: ensuring efficient fat mobilization for energy under hypoxic conditions while establishing sufficient energy reserves to cope with cold and food scarcity.

According to KEGG database analysis, CASTOR2 participates in the mTOR signaling pathway. CASTOR1 and CASTOR2 are highly related proteins that can form homo- or heterodimers and directly interact with GATOR2 to inhibit mTORC1 activity (61). As mentioned in (62), active mTORC1 stimulates biosynthetic pathways including protein, lipid, and nucleotide synthesis while suppressing cellular catabolism through autophagy inhibition, thereby promoting cell growth and proliferation. The upregulation of CASTOR2 contributes to mTORC1 activity inhibition. This inhibitory effect may help reduce unnecessary energy expenditure in high-altitude environments, sharing conceptual similarities with the physiological principle of metabolic flexibility described in (63).

TM4SF18 is enriched in the ferroptosis metabolic pathway. Previous research demonstrates that TM4SF18 upregulation promotes proliferation, migration, and invasion capabilities in gastric cancer (GC) cells (64), while TM4SF18 knockdown effectively suppresses these capacities. Therefore, TM4SF18 downregulation may inhibit proliferation, migration, and invasion capabilities in GC cells.

In summary, close connections exist among ruminal metabolome, small intestinal metabolome, and transcriptome, and their integrated analysis reveals the significant roles these genes play in Bayanbulak sheep’s adaptation to high-altitude cold environments.

## 5 Conclusion

This study demonstrates that Bayanbulak sheep have developed comprehensive adaptations to high-altitude conditions through coordinated physiological changes: Enhanced antioxidant capacity via elevated serum CAT/SOD levels and upregulation of glutathione/purine metabolism pathways, increasing spermidine, L-glutamate and allantoin production; Improved immune function through higher IFN-*γ*/IL-2 levels and AMPK pathway activation, boosting 5’-AMP content; Increased energy reserves via elevated cholesterol/triglycerides/glucose and thermogenesis pathway activation, raising nicotine and oleic acid levels. Multi-tissue analysis revealed significant abundance differences in vanillin, pinacidil and 4-hydroxychalcone across digestive compartments, while gene expression showed ruminal upregulation of CPT1B/CPT1C/CASTOR2 (AMPK/mTOR pathways) and intestinal downregulation of TM4SF18 (ferroptosis pathway). Histologically, thickened epidermis provides additional skin protection. These integrated adaptations collectively enhance oxidative resistance, immune competence and metabolic efficiency in high-altitude environments.

## Data Availability

The data presented in this study are deposited in the Figshare repository (accession number http://doi.org/10.6084/m9.figshare.29551862) and the National Center for Biotechnology Information repository (accession number PRJNA1308259, available at https://www.ncbi.nlm.nih.gov/sra/PRJNA1308259), respectively. For more information regarding our data policies, refer to our guidelines.
